# Pleiotropic Effects of Common and Rare *GCKR* Exonic Mutations on Cardiometabolic Traits

**DOI:** 10.3390/genes13030491

**Published:** 2022-03-10

**Authors:** Kuan-Hung Yeh, Lung-An Hsu, Ming-Sheng Teng, Semon Wu, Hsin-Hua Chou, Yu-Lin Ko

**Affiliations:** 1 Cardiovascular Center and Division of Cardiology, Department of Internal Medicine, Taipei Tzu Chi Hospital, Buddhist Tzu Chi Medical Foundation, New Taipei City 23142, Taiwan; ufddsykh@ms15.hinet.net (K.-H.Y.); chouhhtw@gmail.com (H.-H.C.); 2 School of Medicine, Tzu Chi University, Hualien 97004, Taiwan; 3 The First Cardiovascular Division, Department of Internal Medicine, Chang Gung Memorial Hospital and Chang Gung University College of Medicine, Taoyuan 33305, Taiwan; hsula@cgmh.org.tw; 4 Department of Research, Taipei Tzu Chi Hospital, Buddhist Tzu Chi Medical Foundation, New Taipei City 23142, Taiwan; vincent@tzuchi.com.tw; 5 Department of Life Science, Chinese Culture University, Taipei 11114, Taiwan; semonwu@yahoo.com.tw

**Keywords:** *GCKR* gene, exonic mutation, pleiotropic effect, serum triglyceride level, serum albumin level

## Abstract

Background: The common non-synonymous mutation of the glucokinase regulator (*GCKR*) gene, namely rs1260326, is widely reported to have pleiotropic effects on cardio-metabolic traits and hematological parameters. Objective: This study aimed to identify whether other *GCKR* variants may have pleiotropic effects independent of the rs1260326 genotypes. Methods: In total, 81,097 Taiwan Biobank participants were enrolled for the regional plot association studies and candidate variant analysis of the region around the *GCKR* gene. Results: The initial candidate variant approach showed the significant association of the rs1260326 genotypes with multiple phenotypes. Regional plot association analysis of the *GCKR* gene region further revealed genome-wide significant associations between *GCKR* variants and serum total and low-density lipoprotein cholesterol; triglyceride, uric acid, creatinine, aspartate aminotransferase, γ-Glutamyl transferase, albumin, and fasting plasma glucose levels; estimated glomerular filtration rate; leukocyte and platelet counts; microalbuminuria, and metabolic syndrome, with rs1260326 being the most common lead polymorphism. Serial conditional analysis identified genome-wide significant associations of two low-frequency exonic mutations, rs143881585 and rs8179206, with high serum triglyceride and albumin levels. In five rare *GCKR* exonic non-synonymous or nonsense mutations available for analysis, *GCKR* rs146175795 showed an independent association with serum triglyceride and albumin levels and rs150673460 showed an independent association with serum triglyceride levels. Weighted genetic risk scores from the combination of *GCKR* rs143881585 and rs146175795 revealed a significant association with metabolic syndrome. Conclusion: In addition to the rs1260326 variant, low-frequency and rare *GCKR* exonic mutations exhibit pleiotropic effects on serum triglyceride and albumin levels and the risk of metabolic syndrome. These results provide evidence that both common and rare *GCKR* variants may play a critical role in predicting the risk of cardiometabolic disorders.

## 1. Introduction

The liver is the major organ responsible for both the disposal of oral glucose load (by sensing portal glucose signals, which increase net hepatic glucose uptake) and hypoglycemia (through the initiation of gluconeogenic and glycogenolytic pathways) to maintain healthy blood glucose concentrations [1]. Glucokinase (GCK) is a glucose sensor and is a key regulator of glucose metabolism in the liver through the catalyzation of glucose to glucose-6-phosphate as the first step of glycolysis and through conferring to hepatocytes for cell autonomous regulation in response to plasma glucose fluctuations [2]. Glucokinase regulator protein (GKRP), a hepatocyte-specific inhibitor of GCK, forms the GCK–GKRP complex and acts as a metabolic switch capable of energy storage and activating pathways in response to a period of feeding or fasting [3]. This GKRP-mediated inhibition is associated with nuclear sequestration and GCK inactivation at low glucose concentrations [4]. Additionally, GKRP is regulated by binding to fructose 1-phosphate (F1P) or fructose 6-phosphate (F6P). F1P binding to GKRP reduces GKRP–GCK interactions, whereas F6P enhances such interactions [5]. Paradoxically, GKRP acts as a posttranslational stabilizer of cellular GCK, as indicated by the adenovirus-mediated over-expression of GKRP [6] and *Gckr*-/- mice [7]. GCK bound to GKRP may therefore act as a functional nuclear reserve that can be rapidly activated and mobilized to the cytoplasm following a glucose challenge. Although the exact mechanism is controversial, enhanced glycolytic flux leads to increased triglyceride levels [8].

The glucokinase regulator gene (*GCKR*), a highly pleiotropic gene, encoding GKRP, is located on chromosome 2p23.3 and contains 19 exons and 625 amino acids [9,10]. Recent candidate gene approaches and genome-wide association studies (GWASs) have shown the pleiotropic effects of *GCKR* gene variants in multiple cardiometabolic, biochemical, and hematological pathways [11,12,13,14,15,16,17,18,19,20,21,22,23,24,25,26]. Individuals carrying *GCKR* variants that bind to GCK less effectively are characterized by having low fasting plasma glucose levels and protection from chronic kidney disease; however, this is accompanied by an increased risk of nonalcoholic fatty liver disease, hypertriglyceridemia, hyperuricemia, gout, and metabolic syndrome [17,18,19,20,21,22,23,24,25,26]. Comprehensive fine mapping identified a common non-synonymous variant rs1260326 (p.Pro446Leu) as the likely causative variant associated with an inverse modulation of fasting plasma glucose and serum triglyceride levels [23]. Notably, p.Pro446Leu-GKRP has been shown to attenuate physiologically relevant F6P-mediated inhibition in the formation of the GCK–GKRP complex, reduce nuclear sequestration of GCK, and increase active cytosolic GCK [8,27]. Furthermore, decreased inhibition and sequestration of GCK may lead to increased concentrations of malonyl-CoA, a substrate for de novo lipogenesis, which results in increased triglyceride and cholesterol synthesis and export, as suggested by the associations of *GCKR* with very-low-density lipoprotein (LDL) particle concentrations [8,25]. These results provide a mutational mechanism for the reported association of rs1260326 with increased triglyceride levels and decreased glucose levels.

In addition to the rs1260326, functionally deleterious, rare *GCKR* exonic mutations detected by biochemical and cellular biological assays were collectively associated with hypertriglyceridemia [28]. However, the functional effect of individual variants did not co-segregate with serum triglyceride levels in family studies [29]. Thus, even with functional importance, the critical role of rare exonic *GCKR* mutations in serum triglyceride levels on a population basis remains to be elucidated. By using a candidate variant approach and regional plot association studies with conditional analysis in >80,000 Taiwan Biobank (TWB) participants, this study aimed to test the role of common and rare *GCKR* variants on various metabolic, biochemical, or hematological parameters in Taiwanese. Our data revealed that multiple low-frequency and rare *GCKR* exonic mutations are also significantly associated with serum triglyceride and albumin levels and metabolic syndrome, independent of rs1260326 genotypes.

## 2. Materials and Methods

### 2.1. TWB Cohort

**Figure 1:**
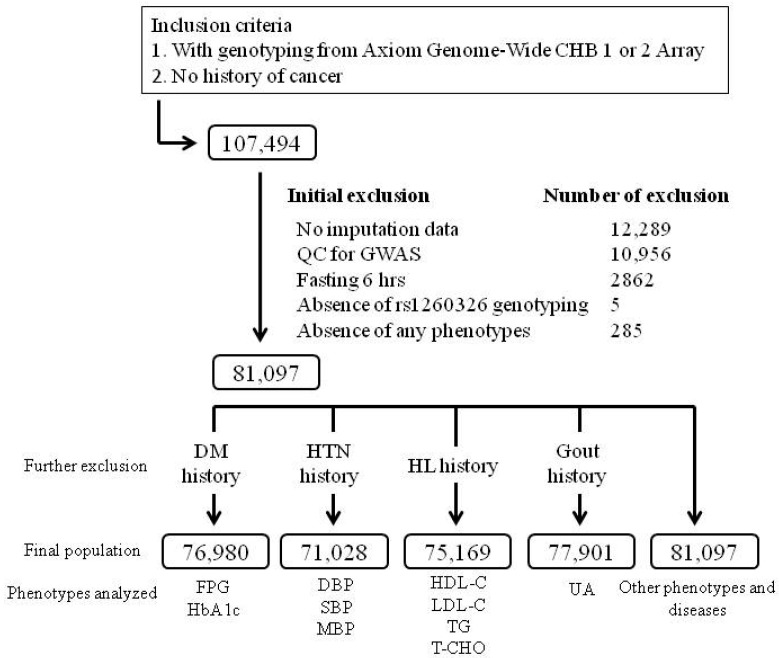
Study flowchart of inclusion and exclusion criteria used to screen Taiwan Biobank project participants. Other phenotypes include age; body mass index; waist circumference; waist–hip ratio; aspartate aminotransferase, alanine aminotransferase, γ-glutamyl transferase, and serum creatinine levels; estimated glomerular filtration rate; serum albumin; total bilirubin; hemoglobin; hematocrit; red blood cell, leukocyte, and platelet counts; and blood urea nitrogen, albuminuria, microalbuminuria, and metabolic syndrome. Abbreviations: QC, quality control; HL, hyperlipidemia; HTN, hypertension; DM, diabetes mellitus; HbA1C, hemoglobin A1C; SBP, systolic blood pressure; DBP, diastolic blood pressure; MBP, mean blood pressure; LDL-C, low-density lipoprotein cholesterol; HDL-C, high-density lipoprotein cholesterol; TG, triglyceride; T-CHO, total cholesterol; UA, uric acid.

The current study cohort was TWB participants recruited from centers across Taiwan between 2008 and 2020. In total, 107,494 participants with no history of cancer, who had GWAS data, were recruited, and 26,397 participants were excluded from the analysis according to the following criteria: no imputation data (12,289 participants), quality control (QC) for the GWAS with identity by descent PI_HAT > 0.187, suggesting 2nd-degree relatives or closer (10,956 participants), fasting for < 6 h (2862 participants), failure of genotyping of the rs1260326 (5), and absence of any study phenotypes (285). The flowchart of participant enrollment is presented in Figure 1. For the analysis of blood pressure status, lipid profiles, glucose metabolism parameters, and serum uric acid level, participants with a history of hypertension, hyperlipidemia, diabetes mellitus, and gout, respectively, were excluded from the analysis. Supplementary Method S1 presents the definitions of hypertension, diabetes mellitus, obesity, current smoking, microalbuminuria, and metabolic syndrome. Ethical approval was received from the Research Ethics Committee of Taipei Tzu Chi Hospital, Buddhist Tzu Chi Medical Foundation (approval number: 05-X04-007) and the Ethics and Governance Council of the TWB (approval number: TWBR10507-02 and TWBR10611-03). Each participant signed an approved informed consent form.

### 2.2. Clinical Phenotypes and Laboratory Examinations

Demographic data used for the analysis included waist circumference, waist–hip ratio, body mass index (BMI), and systolic, mean, and diastolic blood pressure. Biochemistry data used for the analysis included glucose metabolism parameters such as fasting plasma glucose level and hemoglobin A1C; lipid profiles, namely total, high-density lipoprotein (HDL), and low-density lipoprotein (LDL) cholesterol, and triglyceride levels; and liver and renal functional test-related parameters, namely creatinine, uric acid, aspartate aminotransferase (AST), alanine aminotransferase (ALT), γ-glutamyl transferase (γGT), albumin, and total bilirubin levels. BMI and estimated glomerular filtration rate (eGFR) were calculated as previously reported [30]. Hematological parameters analyzed included leukocyte, red blood cell, and platelet counts and hematocrit and hemoglobin levels. Because of the absence of urine creatinine level, only spot urine albumin level was used for urine albumin evaluation.

### 2.3. Selection of GCKR Variants and Genotyping

DNA was isolated from blood samples by using a PerkinElmer chemagic 360 instrument following the manufacturer’s instructions (PerkinElmer, Waltham, MA, USA). SNP genotyping was conducted using custom TWB chips and performed on the Axiom Genome-Wide Array Plate System (Affymetrix, Santa Clara, CA, USA). The *GCKR* variant rs1260326 was initially analyzed, followed by other low-frequency and rare exonic mutations selected for further study (Supplementary Table S1). In this paper, we have used a minor allele frequency (MAF) of <0.01 as rare exonic mutations, as suggested by Wang, et al. [31], and MAF between 0.05 and 0.01 as low-frequency exonic mutations. 

### 2.4. Regional Plot Association Analysis

To identify the lead single-nucleotide polymorphisms (SNPs) around the *GCKR* gene region for various studied phenotypes, we performed a regional plot association analysis by using the data of TWB participants enrolled after QC for GWAS and applying the other exclusion criteria (Figure 1). The Axiom Genome-Wide CHB 1 and 2 Array Plates (Affymetrix, Inc., Santa Clara, CA, USA), comprising 611,656 and 640,160 SNPs, each from 24,927 and 69,529 participants, respectively, were applied for analysis. With 1000 Genomes Project Phase 3 East Asian populations used as a reference panel, genome-wide genotype imputation was performed using SHAPEIT (version 2, Oxford, UK, https://mathgen.stats.ox.ac.uk/genetics_software/shapeit/shapeit.html, accessed on 2 December 2020) and IMPUTE2 (version 2, Oxford, UK, http://mathgen.stats.ox.ac.uk/impute/impute_v2.html, accessed on 2 December 2020). QC was performed after imputation through the filtration of SNPs with IMPUTE2 imputation quality scores of >0.3. Indels were removed using VCFtools (version 0.1, https://vcftools.github.io/index.html, accessed on 2 December 2020). All the samples enrolled for the analysis had a call rate of ≥97%. For SNP QC, the criteria for exclusion from subsequent analyses included an SNP missing rate of <3%, an MAF of <0.01, and a violation of Hardy–Weinberg equilibrium (*p* < 10−6). Finally, 81,097 participants and 139 SNPs were used for regional plot association analysis of the *GCKR* gene region on chromosome 2p23.3 at positions ranging between 27.62 and 27.85 Mb.

### 2.5. Selection of Rare Exonic GCKR Mutations from the Pre-QC Imputation Data for Analysis

During QC for regional plot association analysis, rare variants with MAF of less than 0.01 were excluded. To test for the role of rare exonic mutations in genotype–phenotype associations, we selected variants from the pre-QC imputation data for analysis. A total of five rare exonic *GCKR* mutations were enrolled for the genotype–phenotype association analysis, including four non-synonymous mutations (rs146175795, p.Val103Met; rs150673460, p.Pro132Leu; rs1414321043, Ala314Ser; and rs146285804, p.Trp517Cys) and one nonsense mutation (rs149847328, p.Arg227Ter) (Supplementary Table S1).

### 2.6. Statistical Analysis

Continuous variables were expressed as mean ± standard deviation. When the distribution was strongly skewed, median and interquartile ranges were given, which were tested using a two-sample t test or analysis of variance. Differences in categorical data distribution were examined using a chi-squared test or chi-squared test for trend. Before analysis, all study parameters were logarithmically transformed to adhere to a normality assumption. We assumed the genetic effect to be additive after adjustment for age, sex, BMI, and current smoking status, and a general linear model was used to analyze the studied phenotypes in relation to the predictors of investigated genotypes and confounders. Regional plot association studies with conditional analysis were conducted using the analysis software package PLINK (version 1.07, Shaun Purcell, Cambridge, MA, USA, https://zzz.bwh.harvard.edu/plink/, accessed on 14 August 2021). Conditional analysis is a tool to identify secondary association signals at a candidate locus by adjusting the index variant in the region [32,33]. For the regional plot association study, conditional analysis was conducted by testing the residual association with all remaining SNPs, and another round of conditional analysis can be performed by adjusting the second index variant in this region for independent signals. Genome-wide significance was defined by *p* < 5 × 10^−8^. For Bonferroni correction of regional plot associational analysis, the significant value was defined as *p* < 1.0 × 10^−5^, calculated as 0.05/(139 × 33), according to a total of 139 variants and 33 traits analyzed. For Bonferroni correction of each genotype–phenotype analysis with rare mutations, we used a more liberal threshold of *p* < 2.9 × 10^−4^, calculated as 0.05/5 × 33, according to a total of 5 rare variants and 33 traits analyzed. For weighted genetic risk score (WGRS), we weighted the SNPs in each allele score by using the β coefficients from our association analysis, and the risk allele was selected with directionally concordant associations of target parameters. The LDmatrix (https://analysistools.nci.nih.gov/LDlink/?tab=ldmatrix, accessed on 19 April 2021) was used for the analysis of linkage disequilibrium (LD). SPSS (version 22; SPSS, Chicago, IL, USA) was used to perform all calculations.

## 3. Results

### 3.1. Association of GCKR rs1260326 Genotypes with Clinical, Metabolic, and Biochemical Phenotypes and Hematological Parameters

The data of the *GCKR* exonic mutations enrolled for the analysis are presented in Supplementary Table S1. In total, >80,000 volunteers participated in the genotype–phenotype association analysis (Table 1). By using an additive model, after adjustment of age, sex, BMI, and smoking status, genome-wide significant associations were found for rs1260326 genotypes with total and LDL cholesterol and triglyceride levels, fasting plasma glucose, serum uric acid levels, renal functional parameters (serum creatinine and urine albumin levels and eGFR), liver functional parameters (AST, γGT, and serum albumin), and leukocyte and platelet counts, whereas associations with *p* < 1.0 × 10^−5^ were noted for systolic and mean blood pressure. The C allele of the rs1260326 variant is associated with higher systolic and mean blood pressure; serum total and LDL cholesterol, triglyceride, uric acid, AST, γGT, and albumin levels; eGFR; and leukocyte and platelet counts, and lower fasting plasma glucose and serum creatinine levels.

### 3.2. Association of GCKR rs1260326 Genotypes with Risk Factors for Atherosclerosis

We analyzed the association of rs1260326 genotypes with atherosclerotic risk factors. In our study, after adjustment for sex, age, BMI, and current smoking status, hypertension, microalbuminuria, and metabolic syndrome were found to be significantly associated with rs1260326 genotypes (Table 2). The C allele of the rs1260326 genotype is associated with a higher risk of hypertension, microalbuminuria, and metabolic syndrome.

### 3.3. Regional Plot Association Studies for Determining the Associations of Genetic Variants at Positions 27.62 to 27.85 Mb on Chromosome 2p23.3 with Study Phenotypes

Regional plot association analyses were performed to determine the association of genetic variants around the *GCKR* gene region at positions 27.62–27.85 Mb on chromosome 2p23.3 with study phenotypes. Our data revealed that the lead SNP for each phenotype was situated at or near the *GCKR* gene region, revealing pleiotropic effects on this gene locus (Figure 2, Supplementary Figures S1–S20). Regional plot association analysis showed genome-wide significant associations between *GCKR* variants and serum total and LDL cholesterol; triglyceride, uric acid, creatinine, AST, γ-GT, albumin, and fasting plasma glucose levels; eGFR; urine albumin levels, and leukocyte and platelet counts, microalbuminuria, and metabolic syndrome (Table 3). 

### 3.4. Linkage Disequilibrium between GCKR Gene Region SNPs

**Figure 3:**
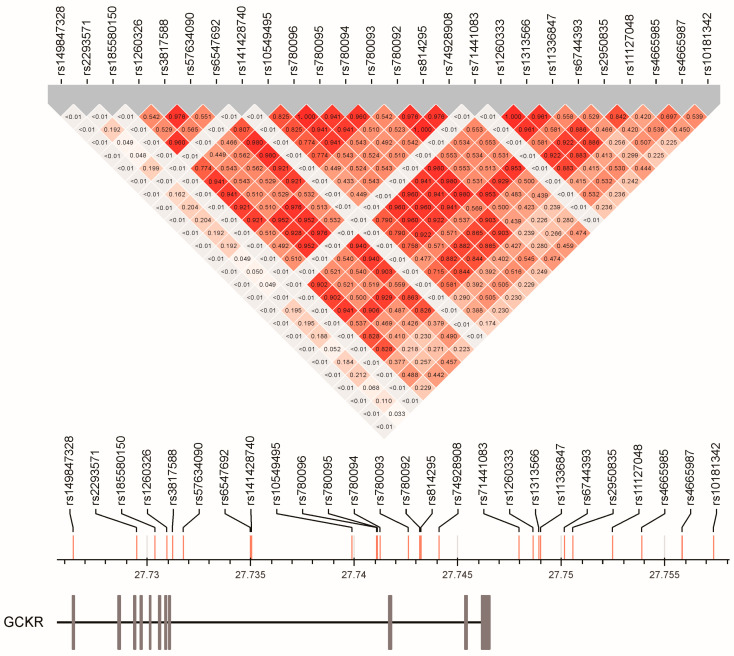
Linkage disequilibrium map of *GCKR* gene region single-nucleotide polymorphisms.

The rs1260326 was the most common lead SNP for lipid profile, blood pressure status, fating plasma glucose, serum uric acid, albumin, and AST levels, urine albumin levels, and metabolic syndrome (Table 3). Most of the other lead SNPs had a strong LD with the rs1260326 variant (all r^2^ > 0.82), whereas for the phenotypes of leukocyte count and serum alanine aminotransferase (ALT) levels, the lead SNPs rs6744393 and rs12989678, respectively, had a moderate LD with the rs1260326 variant (r^2^ = 0.537 and r^2^ = 0.476, respectively; Figure 3). Further, after serial conditional analysis was conducted, the lead SNPs rs143881585 and rs8179206 for serum triglyceride and albumin levels showed a weak LD with the lead SNPs (all r^2^ < 0.015 for the rs143881585 variant and maximal r^2^ = 0.029 for the rs8179206 variant). The linkage disequilibrium map of the *GCKR* variants is presented in Figure 3.

### 3.5. Association of GCKR rs143881585 and rs8179206 Genotypes with Clinical, Metabolic, and Biochemical Phenotypes, Hematological Parameters, and Risk Factors for Atherosclerosis

**Figure 4:**
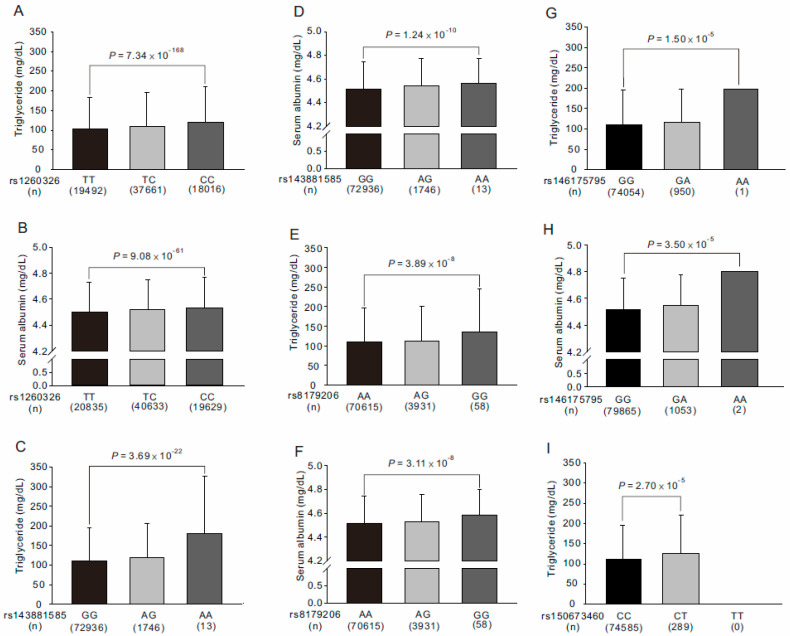
Association of *GCKR* exonic mutations with serum triglyceride (**A**,**C**,**E**,**G**,**I**) and albumin (**B**,**D**,**F**,**H**) levels in Taiwan Biobank project participants. Further adjusted for rs1260326 genotypes in conditional analysis (**C**,**D**). Further adjusted for rs1260326 and rs143881585 genotypes in conditional analysis (**E**,**F**).

We further tested the association of rs143881585 and rs8179206 genotypes with the study phenotypes (Supplementary Tables S2–S5). After adjustment for age, sex, current smoking, and BMI, our data revealed that individuals with the A and G alleles of the rs143881585 and rs8179206 variants, respectively, had significantly higher serum triglyceride and albumin levels (*p* = 1.17 × 10^−10^ and *p* = 7.00 × 10^−6^, respectively, for the rs143881585 variant and *p* = 0.0046 and *p* = 1.19 × 10^−4^, respectively, for the rs8179206 variant; Figure 4). The associations of the rs143881585 genotypes became more significant after further adjustment of the rs1260326 genotypes (*p* = 3.69 × 10^−22^ and *p* = 1.24 × 10^−10^, respectively, for serum triglyceride and albumin levels; Supplementary Tables S2 and S3 and Figure 4C,D). With further adjustment of both rs1260326 and rs143881585 genotypes for the conditional analysis, genome-wide significant associations were noted between the G allele of the rs8179206 variant and higher serum triglyceride and albumin levels (*p* = 3.89 × 10^−8^ and *p* = 3.11 × 10^−8^, respectively; Supplementary Tables S4 and S5 and Figure 4E,F).

### 3.6. Association between Rare GCKR Exonic Mutations and Clinical Phenotypes and Laboratory Parameters

From the pre-QC imputation data, five rare exonic nonsynonymous or nonsense mutations were selected for genotype–phenotype association analysis (Supplementary Tables S6–S15). All the rare *GCKR* mutations showed a weak LD with the other genotypes (r^2^ < 0.01) (Supplementary Figure S21). After adjustment for age, sex, body mass index (BMI), and current smoking, in genotype–phenotype association analysis, rs146175795 genotypes showed significant associations with serum triglyceride and albumin levels (*p* = 1.50 × 10^−5^ and *p* = 3.50 × 10^−5^, respectively, Supplementary Tables S8 and S9, Figure 4G,H) and rs150673460 genotypes showed significant associations with serum triglyceride levels (*p* = 2.70 × 10^−5^; Supplementary Table S10, Figure 4I). 

### 3.7. Stepwise Linear Regression Analysis for Serum Triglyceride and Albumin Levels

A stepwise linear regression analysis using age, sex, body mass index, current smoking, and *GCKR* variants revealed that rs1260326, rs143881585, rs8179206, rs146175795, and rs150673460 genotypes contributed to 0.83%, 0.10%, 0.05%, 0.03%, and 0.01% of the variation in serum triglyceride levels and that rs1260326, rs143881585, rs8179206, and rs146175795 genotypes contributed to 0.32%, 0.05%, 0.04%, and 0.04% of the variation in serum albumin levels, respectively (Table 4).

### 3.8. WGRS from the Combination of GCKR rs143881585 and rs1461755795 Revealed Significant Association with Metabolic Syndrome

In addition to rs1260326, the low-frequency and rare *GCKR* variants rs143881585 and rs146175795 were linked to metabolic syndrome, but the *p* values (0.0402 and 0.0005, respectively) fell short of the statistical significance cut-off (1.0 × 10^−5^). We wanted to see if the combination of these two variations had an influence on metabolic syndrome risk. We discovered that the combined low-frequency and rare variants rs143881585 and rs146175795 in the *GCKR* gene are highly linked with the risk of metabolic syndrome (*p* = 1.83 × 10^−6^) using WGRS from rs143881585 and rs146175795 variants (Supplementary Table S16).

## 4. Discussion

**Figure 5:**
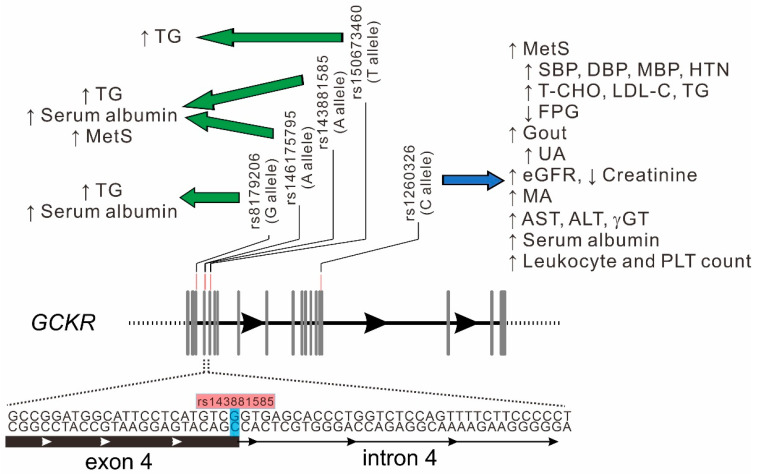
Genomic structure of *GCKR* variants and their association with various phenotypes/diseases. Green arrow: association with low-frequency and rare *GCKR* variants. Blue arrow: association with rs1260326 genotypes. Abbreviations: MetS, metabolic syndrome; MA, microalbuminuria; PLT, platelet. Other abbreviations as in Figure 1 and Table 2.

In this investigation, the combined approaches of analyzing candidate gene variants and a regional plot association study were used to confirm the pleiotropic effect of the rs1260326 genotypes on multiple quantitative traits and diseases. Through regional plot association analysis, all lead SNPs showed moderate to strong LD with the rs1260326 variant. With serial conditional analysis, we demonstrated novel genome-wide significant associations of *GCKR* exonic mutations rs143881585 and rs8179206 with serum triglyceride and albumin levels. With further rare *GCKR* exonic mutation analysis, *GCKR* rs146175795 also showed a significant association with serum triglyceride and albumin levels and rs150673460 showed an independent association with serum triglyceride levels. In combination, the aforementioned variants contributed to 1.02% and 0.45% of serum triglyceride and albumin levels, respectively. All these results showed the same direction of elevated serum triglyceride and albumin levels with mutation alleles (Figure 5). We then tested the effect of combination of the low-frequency and rare *GCKR* variants by using WGRS analysis and found a significant association with metabolic syndrome. To our knowledge, this is the first report to describe novel associations of multiple low-frequency and rare *GCKR* variants with serum triglyceride and albumin levels and metabolic syndrome independent of the rs1260326 variant. With the combination of candidate variants and regional plot association studies, these results may provide further evidence for understanding the critical role of the *GCKR* gene in the risk of cardiometabolic disorders.

### 4.1. Pleiotropic Effect of GCKR Gene Locus

Kanai et al. [16] showed that the *GCKR* region is one of the most pleiotropic regions and is associated with 18 quantitative traits. The NHGRI-EBI GWAS Catalog is a publicly available resource for GWASs, and it contains useful visualizations of variant–trait associations. All variants are mapped onto chromosomal positions on the human genome (https://www.ebi.ac.uk/gwas, accessed on 2 December 2020). When the data of the GWAS Catalog were reviewed, genome-wide significant associations with the *GCKR* region variants were noted, with the associations including various demographic factors (body height, BMI, lean body mass, and heart rate), cardiometabolic traits, liver and kidney functional tests and diseases, lifestyle factors (such as alcohol consumption, coffee consumption, and dietary factors), biomarker and hormone levels (such as C reactive protein, leptin, YKL40, testosterone, and estradiol levels), hematological parameters, coagulation factors, electrolyte and metabolite levels, and various diseases (such as gallstone, urolithiasis, and age-related diseases). Moreover, our data revealed the association of *GCKR* gene variants with multiple quantitative traits and diseases, in which 19 of them have genome-wide significance (*p* < 5 × 10^−8^). All these data support the notion that the *GCKR* gene and its encoded protein GKRP are involved in the functions of multiple organs and systems, revealing its critical role in mediating homeostasis in the human body. In addition to the pleiotropic effect of the *GCKR* gene locus, our findings confirmed a recent finding in a Korean population that many cardiometabolic traits had a common genetic foundation [34]. The relevance of understanding pleiotropy and its consequences for genetic testing and personal genomics has been highlighted by the expanding use of genetic information in clinical practice.

### 4.2. Bidirectional Effects of GCKR rs1260326 Variant on Associated Phenotypes

With the pleiotropic effect of *GCKR* variants, the allelic effects of SNPs conferred both favorable and unfavorable outcomes on the human body. As previously reported [17,18,19,20,21,22,23,24,25,26], our data revealed that the C allele carrier of the rs1260326 variant is associated with multiple unfavorable phenotypes, such as higher systolic, diastolic, and mean BP and risk of hypertension and microalbuminuria, elevated levels of liver function indicators (such as serum ALT, AST, and γGT levels), higher total and LDL cholesterol and triglyceride and uric acid levels, as well as a higher risk of metabolic syndrome and gout. Furthermore, the C allele carrier of the rs1260326 variant is associated with preferable phenotypes, such as a lower fasting plasma glucose level and higher eGFR and serum albumin levels. These results are consistent with those reported previously [17,18,19,20,21,22,23,24,25] and the diverse and bidirectional effects of the rs1260326 variant reveal that a more cautious approach should be considered when *GCKR* is used as a target of drug therapy.

### 4.3. Association between the rs143881585 Variant and Serum Triglyceride and Albumin Levels Is Independent of the rs1260326 Variant

The rs143881585 variant is a low-frequency one in the Taiwanese population, with a minor allele frequency (MAF) of 1.19% in TWB project participants. According to the PUBMed.gov website, the allele frequency of rs143881585 was 0% in 1006 participants from the 1000 Genomes project and 0.102% in 60,706 unrelated individuals from The Exome Aggregation Consortium, with exome sequencing data obtained from diverse large-scale sequencing projects. These results suggest that rs143881585 may be a rare variant in other populations. Furthermore, the association of rs143881585 genotypes with serum triglyceride and albumin levels was partially suppressed by rs1260326, with the association becoming stronger after adjustment for rs1260326 genotypes. In addition, rs143881585 is a synonymous variant (c.354 G > A, p.Ser118Ser) that lies at the branch point between exon 4 and intron 5. Whether this variant affects gene splicing is unknown. Thus, a further mechanistic study may be necessary to elucidate the molecular basis of the rs143881585 variant’s association with serum triglyceride and albumin levels.

### 4.4. Association of the rs8179206 Variant with Serum Triglyceride and Albumin Levels Is Independent of the rs1260326 Variant

The SNP rs8179206 (c. 296 A to G, p.Glu77Gly), a previously reported, rare *GCKR* variant, is a non-synonymous variation located on exon 3 of the *GCKR* gene [28,29]. From the dbSNP database, the MAFs for European Descent participants of 1000 Genomes Phase V1, African, and East Asian populations were 0%, 0%, and 2.3%, respectively, suggesting that this variant is rare in non-East Asian populations. In our study, the frequency of minor allele rs8179206 was 2.78%, similar to those previously reported in East Asian populations, including Chinese [35]. In terms of function, the rs8179206 variant was classified as a putative loss-of-function mutation with reduced nuclear localization of GKRP, GKRP expression, and F6P binding [28,29]. Chinese studies with a relatively small sample size have shown no significant association of SNP rs8179206 with obesity, type 2 diabetes mellitus, and various lipid profiles [35,36,37]. With >80,000 TWB project participants for analysis, we provide the first evidence regarding the genome-wide association of this variant with serum triglyceride and albumin levels.

### 4.5. Role of Rare GCKR Exonic Mutations in Serum Triglyceride Levels 

Rare nonsynonymous variants in the *GCKR* gene have been associated with variations in metabolic traits, particularly serum triglyceride levels [28,29,38]. A recent study highlighted the clinical relevance of the collective burden of rare alleles in *GCKR*, reporting that non-synonymous variants with an MAF of <0.01 in the *GCKR* gene are enriched in cases of extreme hypertriglyceridemia [38]. When exonic Sanger sequencing of the *GCKR* gene in 800 individuals (mostly of non-Hispanic mixed European descent) was applied, 19 nonsynonymous rare *GCKR* variants were detected, which, in combination, showed significantly higher serum triglyceride levels compared to those without such variants [28]. However, rare loss-of-function *GCKR* variants do not co-segregate with increased plasma triglyceride levels in families [29]. By contrast, in two South Asian population-specific, functionally disruptive, rare *GCKR* non-synonymous mutations, rs774930016 (p.Ser105Asn) and rs55537970 (p.Arg553Trp), significantly increased risks of hypertriglyceridemia have been reported [39]. Rees et al. [28] subdivided rare *GCKR* variants into three classes according to cellular localization, cellular interaction with GCK, and kinetic effects. Several reports have shown *GCKR* rs146175795 (p.Val103Met) in patients with hypertriglyceridemia [28,40,41,42]. The *GCKR* p.Val103Met mutation was predicted to be disease-causing by in silico algorithms (SIFT, Poly-Phen-2, and FATHMM). The rs146175795 variant was also classified as a putative loss-of-function mutation with reduced nuclear localization of GKRP, GKRP expression, and F6P binding [28]. Jin et al. [40] performed genetic analysis for 103 Chinese patients with very high triglyceride levels and found that 46 patients had rare pathogenic/potential pathogenic variants in 15 triglyceride-related genes, in which four of them had the *GCKR* p.Val103Met mutation. The MAF of the *GCKR* p.Val103Met mutation has been shown to be <0.001 in European and African populations and 0.0069 in East Asian populations according to the PUBMed.gov website. In our study participants, the MAF was 0.0065. In addition to serum triglyceride levels, our results also showed a genome-wide significant association of *GCKR* p.Val103Met mutation with serum albumin levels. One non-synonymous exonic mutation, rs150673460, has not been previously reported and was found to be associated with serum triglyceride levels in our study cohort. 

### 4.6. GCKR Variants and Serum Albumin Levels

Previous GWAS studies have shown *GCKR* as a candidate gene locus for serum albumin levels, with rs1260326 as the lead SNP [13,43,44]. Our data also revealed the consistent association of several low-frequency and rare *GCKR* variants with elevated serum albumin levels. Albumin is a multifaced protein synthesized in the hepatocytes that has many physiological properties, including anti-inflammatory, antioxidant, anticoagulant, and anti-platelet aggregation activities, and serum albumin has been assigned as an indicator of malnutrition [45]. In a meta-analysis of more than one million apparently healthy adult participants, elevated serum albumin levels were associated with a reduced risk of hypertension, adverse vascular events, all-cause mortality, certain cancers, and fracture, revealing serum albumin as a biomarker in determining the risk of adverse cardiometabolic outcomes [46]. Using a GWAS study, Loomis et al. [47] further showed that *GCKR* rs1260326 genotypes are significantly associated with percent glycated albumin, which is in the inverse direction of the association with serum albumin levels. Glycated albumin, a ketoamine formed by the non-enzymatic glycation of serum albumin, is calculated as the percentage of serum albumin that is in glycated form. A significant and negative correlation was found between glycated albumin and serum albumin levels [48], and the serum glycated albumin value decreased by 0.23% with every 1g/dL increase in serum albumin [49]. Glycated albumin has been suggested to be an alternative to HbA1C in situations when HbA1C is not recommended for plasma glucose monitoring, such as hemoglobinopathies, pregnancy, or chronic kidney disease [50,51,52]. Increase glycated albumin values have been associated with diabetic nephropathy, neuropathy, and cardiovascular complications [53]. With increased serum albumin levels, the *GCKR* variants may also decrease the glycated albumin values. Thus, the *GCKR* variants may affect the cardiometabolic outcomes not only by increasing serum albumin levels but also by decreasing glycated albumin values. Further studies may help to elucidate more of the potential roles for the *GCKR* variants in serum albumin levels, serum glycated albumin values, and cardiometabolic outcomes.

### 4.7. Association between GCKR Variants and Metabolic Syndrome

Previous studies have focused on gene–environmental factor (obesity or diet) interactions explaining ‘missing heritability’ for phenotypic variance in MetS risk [54,55], accounting for strong but paradoxical relationships of *GCKR* polymorphisms with lower fasting plasma glucose and higher TG levels among MetS components. This study looked into other possibilities, such as significantly larger numbers of variants with lower effects that have yet to be discovered or rarer variants (perhaps with larger impacts) that are poorly detected by existing genotyping arrays. Because four low-frequency and rare *GCKR* variants have a strong and persistent connection with serum triglyceride levels, aggregation of their effects may increase the risk of metabolic syndrome independently of the established risk of common polymorphism rs1260326. Our findings, by the combination of *GCKR* variants rs143881585 and rs1461755795, add to the growing body of evidence that GSKR plays a critical role in the development of metabolic syndrome.

## 5. Conclusions

Our investigation confirmed the pleiotropic effect of the *GCKR* variants, particularly the *GCKR* rs1260326. Moreover, multiple low-frequency and rare *GCKR* variants were found to contribute to various aspects of cardiometabolic traits, such as serum triglyceride and albumin levels and metabolic syndrome, in this Taiwanese population, independent of the rs1260326 variant. A larger population study with meta-analysis and trans-ethnic population analysis may help to further elucidate the critical role of *GCKR* variants in cardiometabolic traits and diseases.

## Figures and Tables

**Figure 2:**
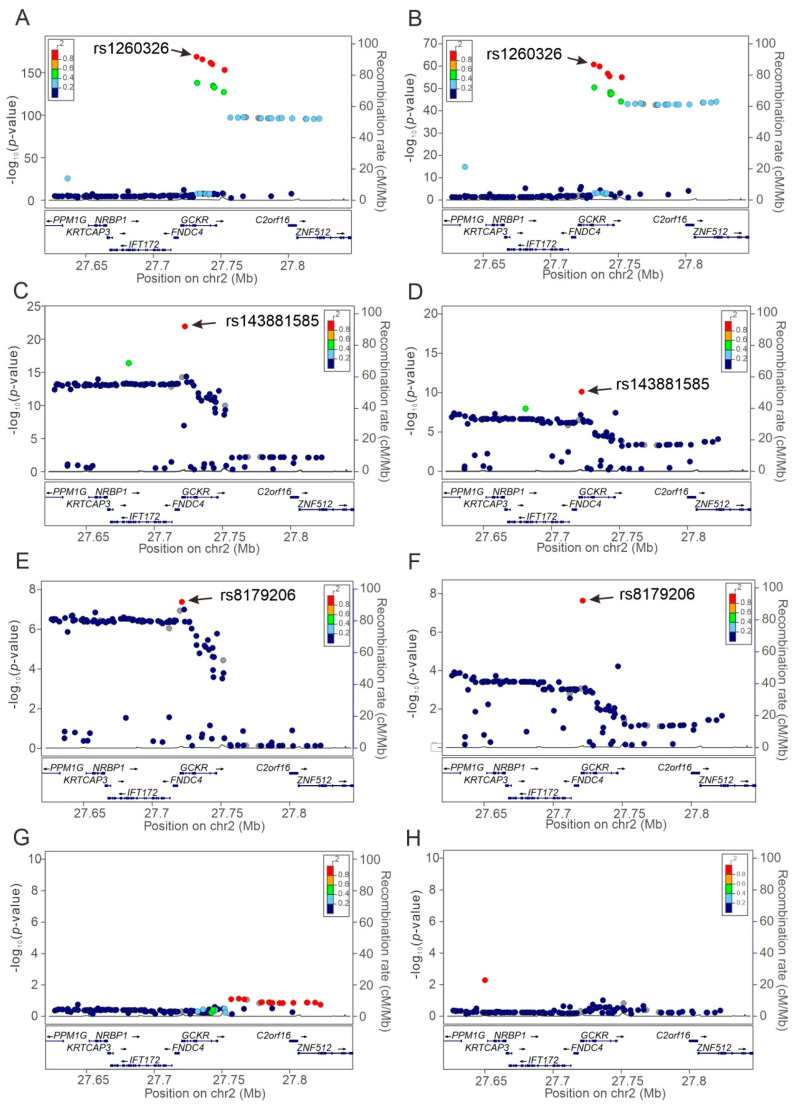
Regional plot associations of the *GCKR* gene region for the serum triglyceride and albumin levels. Regional plot associations are shown without (**A**,**B**) or with serial conditional analysis after further adjustment for rs1260326 (**C**,**D**), rs143881585 (**E**,**F**), and rs8179206 (**G**,**H**) genotypes. Association analyses were performed for serum triglyceride (**A**,**C**,**E**,**G**) and albumin (**B**,**D**,**F**,**H**) levels. *GCKR*, glucokinase regulator.

**Table 1:** Association of the rs1260326 genotypes with metabolic and hematological phenotypes.

Clinical and Laboratory Parameters	Total	β	SE	*p* Value *
Anthropology				
Age (years)	51.0 (41.0–59.0)	0.0767	0.0530	0.1481
Waist circumference (cm)	83.0 (76.0–89.5)	0.0533	0.0253	0.0350
Waist–hip ratio	0.87 ± 0.07	0.0002	0.0003	0.3493
Body mass index (kg/m^2^)	23.8 (21.6–26.3)	−0.0318	0.0180	0.0773
Blood pressure				
Systolic BP * (mmHg)	115.0(105.0–127.0)	0.3931	0.0762	2.48 × 10^−7^
Diastolic BP * (mmHg)	71.0 (65.0–79.0)	0.2182	0.0497	1.10 × 10^−5^
Mean BP * (mmHg)	86.0 (78.7–94.3)	0.2765	0.0546	4.08 × 10^−7^
Lipid profiles				
Total cholesterol # (mg/dL)	171.0 (193.0–216.0)	0.0052	0.0004	1.74 × 10^−39^
HDL cholesterol # (mg/dL)	53.0 (45.0–63.0)	−0.0003	0.0005	0.5755
LDL cholesterol # (mg/dL)	119.0 (99.0–140.0)	0.0047	0.0006	1.90 × 10^−15^
Triglyceride # (mg/dL)	90.0 (63.0–132.0)	0.0302	0.0011	7.34 × 10^−168^
Glucose metabolism				
Fasting plasma glucose ** (mg/dL)	92.0 (87.0–97.0)	−0.6133	0.0750	2.83 × 10^−16^
HbA1C ** (%)	5.6 (5.4–5.9)	−0.0038	0.0030	0.2031
Uric acid				
Uric acid *** (mg/dL)	5.2 (4.4–6.2)	0.0714	0.0055	4.72 × 10^−38^
Renal function				
Creatinine (mg/dL)	0.68 (0.57–0.83)	−0.0072	0.0011	9.45 × 10^−12^
eGFR (mL/min/1.73 m^2^)	100.7 (87.5–116.4)	1.1035	0.1074	9.07 × 10^−25^
Urine albumin (mg/L)	8.7 (5.4–15.2)	0.0147	0.0023	9.64 × 10^−11^
Liver function				
AST (U/L)	23.0 (20.0–27.0)	0.3812	0.0602	2.43 × 10^−10^
ALT (U/L)	19.0 (14.0–27.0)	0.3921	0.0929	2.40 × 10^−5^
γGT (U/L)	17.0 (12.0–26.0)	1.3605	0.1550	1.68 × 10^−18^
Serum albumin (g/dL)	4.5 (4.4–4.6)	0.0182	0.0011	9.08 × 10^−61^
Total bilirubin (mg/dL)	0.6 (0.5–0.8)	0.0022	0.0013	0.0968
Hematological parameters				
Leukocyte count (10^3^/μL)	5.7 (4.7–6.8)	0.0523	0.0076	8.17 × 10^−12^
Hematocrit (%)	41.6 (39.0–44.5)	−0.0387	0.0173	0.0256
Platelet count (10^3^/μL)	237.0 (202.0–276.0)	2.0649	0.2833	3.17 × 10^−13^
Red blood cell count (10^6^/μL)	4.7 (4.4–5.0)	−0.0052	0.0022	0.0189
Hemoglobin (g/dL)	13.7 (12.8–14.8)	−0.0118	0.0061	0.0545

*p*: adjusted for age, BMI, and current smoking; Age: adjusted for BMI and current smoking; and BMI: adjusted for age and smoking. Participants were analyzed after the exclusion of those with a history of * hypertension, ** diabetes mellitus, *** gout, and # hyperlipidemia. Data are presented as median (interquartile range). Abbreviations: SE, standard error; BP, blood pressure; HDL, high-density lipoprotein; LDL, low-density lipoprotein; HbA1C, hemoglobin A1C; eGFR, estimated glomerular filtration rate; BUN, blood urea nitrogen; AST, aspartate aminotransferase; ALT, alanine aminotransferase; γGT, γ-glutamyl transferase; BMI, body mass index.

**Table 2:** Association between rs1260326 genotypes and atherosclerotic risk factors.

Genotypes	TT	TC	CC	β	SE	*p* Value *
Diabetes mellitus (%)	9.8	9.4	9.4	−0.0284	0.0178	0.1105
Hypertension (%)	21.6	22.3	23.4	0.0639	0.0132	1.00 × 10^−6^
Current smoking (%)	9.2	9	9.2	0.0156	0.0182	0.3908
Gout (%)	3.7	3.8	4.5	0.1153	0.0266	1.40 × 10^−5^
Microalbuminuria (%)	10.6	11.2	12.4	0.0953	0.0159	2.16 × 10^−9^
Metabolic syndrome (%)	23.9	24.8	26.2	0.0884	0.0133	2.51 × 10^−11^

* *p* value adjusted for age, sex, body mass index, and current smoking. Current smoking: adjusted for age, BMI, and sex. Abbreviation: SE, standard error.

**Table 3:** Lead single-nucleotide polymorphisms at the *GCKR* gene region.

Phenotypes	Lead SNPs	*p* Value	Position	Allele #	MAF	LD ##	Function	Amino Acid (Codon)
Triglyceride (mg/dL)	rs1260326	7.34 × 10^−168^	27508073	T/C	0.4997	1	Missense variant	Pro446Leu
	rs143881585 *	3.69 × 10^−22^	27498323	G/A	0.0132	<0.015	Synonymous Variant	Ser118Ser
	rs8179206 **	3.89 × 10^−8^	27497575	A/G	0.0271	0.029	Missense variant	Glu77Gly
Serum albumin (mg/L)	rs1260326	9.08 × 10^−61^	27508073	T/C	0.4997	1	Missense variant	Pro446Leu
	rs143881585 *	1.24 × 10^−10^	27498323	G/A	0.0132	<0.015	Synonymous Variant	Ser118Ser
	rs8179206 **	3.11 × 10^−8^	27497575	A/G	0.0271	0.029	Missense variant	Glu77Gly
Systolic BP (mmHg)	rs1260326	2.48 × 10^−7^	27508073	T/C	0.4997	1	Missense variant	Pro446Leu
Diastolic BP (mmHg)	rs1260326	1.10 × 10^−5^	27508073	T/C	0.4997	1	Missense variant	Pro446Leu
Mean BP (mmHg)	rs1260326	4.08 × 10^−7^	27508073	T/C	0.4997	1	Missense variant	Pro446Leu
Total cholesterol (mg/dL)	rs1260326	1.74 × 10^−39^	27508073	T/C	0.4997	1	Missense variant	Pro446Leu
LDL cholesterol (mg/dL)	rs1260326	1.90 × 10^−15^	27508073	T/C	0.4997	1	Missense variant	Pro446Leu
Fasting plasma glucose (mg/dL)	rs1260326	2.83 × 10^−16^	27508073	T/C	0.4997	1	Missense variant	Pro446Leu
Uric acid (mg/dL)	rs1260326	4.72 × 10^−38^	27508073	T/C	0.4997	1	Missense variant	Pro446Leu
Creatinine (mg/dL)	rs2950835	9.45 × 10^−12^	27527678	A/G	0.5040	0.828	Downstream gene variant	--
eGFR (mL/min/1.73 m^2^)	rs2950835	9.07 × 10^−25^	27527678	A/G	0.5040	0.828	Downstream gene variant	--
Urine albumin (mg/L)	rs1260326	9.64 × 10^−11^	27508073	T/C	0.4997	1	Missense variant	Pro446Leu
AST (U/L)	rs1260326	2.43 × 10^−10^	27508073	T/C	0.4997	1	Missense variant	Pro446Leu
ALT (U/L)	rs12989678	2.40 × 10^−5^	27598615	C/T	0.4935	0.476	Intron variant	--
γGT (U/L)	rs780093	1.68 × 10^−18^	27519736	T/C	0.4941	0.921	Intron variant	--
Leukocyte counts (10^3^/μL)	rs6744393	8.17 × 10^−12^	27527272	C/T	0.3524	0.537	Downstream gene variant	--
Platelet counts (10^3^/μL)	rs6547692	3.17 × 10^−13^	27512105	G/A	0.4944	0.960	Intron variant	--
Hypertension	rs2950835	1.00 × 10^−6^	27527678	A/G	0.5040	0.828	Downstream gene variant	--
Gout	rs780094	1.40 × 10^−5^	27518370	T/C	0.5099	0.921	Intron variant	--
Microalbuminuria	rs6547692	2.16 × 10^−9^	27512105	G/A	0.4944	0.960	Intron variant	--
Metabolic syndrome	rs1260326	2.49 × 10^−11^	27508073	T/C	0.4997	1	Missense variant	Pro446Leu

* After conditional analysis adjusting for rs1260326. ** After conditional analysis adjusting for rs1260326 and rs143881585. # Allele: Reference allele/alternate allele. ## LD with the rs1260326 variant. Abbreviations: *GCKR*, glucokinase regulator; BP, blood pressure; LDL, low-density lipoprotein; eGFR, estimated glomerular filtration rate; BUN, blood urea nitrogen; AST, aspartate aminotransferase; ALT, alanine aminotransferase; GGT, γ-glutamyl transferase; MAF, minor allele frequency; LD, linkage disequilibrium; SNV, single-nucleotide variation.

**Table 4:** Serum triglyceride level and serum albumin level: stepwise linear regression analysis, including genotypes.

	Serum Triglyceride Level (75,169 *)			Serum Albumin Level (81,097)		
	β	r^2^	*P*	β	r^2^	*P*
Age (years)	0.0031	0.0175	<10^−307^	−0.0034	0.0243	<10^−307^
Sex (male vs. female)	−0.0560	0.0189	8.11 × 10^−226^	−0.1179	0.0503	<10^−307^
Body mass index (kg/m^2^)	0.0223	0.1475	<10^−307^	−0.0033	0.0028	1.23 × 10^−52^
Current smoking (%)	0.0819	0.0090	6.70 × 10^−181^	−0.0202	0.0005	2.47 × 10^−12^
rs1260326 (TT vs. TC vs. CC)	0.0328	0.0083	1.55 × 10^−188^	0.0205	0.0032	1.58 × 10^−73^
rs143881585 (GG vs. GA vs. AA)	0.0499	0.0010	1.11 × 10^−22^	0.0344	0.0005	2.14 × 10^−11^
rs146175795 (GG vs. GA)	0.0474	0.0005	8.36 × 10^−12^	0.0401	0.0004	6.83 × 10^−9^
rs8179206 (AA vs. AG vs. GG)	0.0190	0.0003	2.13 × 10^−8^	0.0194	0.0004	1.60 × 10^−8^
rs150673460 (CC vs. CT)	0.0401	0.0001	0.0013			

* Participants were analyzed after the exclusion of those with a history of hyperlipidemia.

## Data Availability

The data presented in this study are available on request from the corresponding author.

## Supplementary Materials

The following supporting information can be downloaded at: https://www.mdpi.com/article/10.3390/genes13030491/s1. Method S1: Definitions of hypertension, diabetes mellitus, obesity, current smoking, microalbuminuria and metabolic syndrome. Table S1: *GCKR* exonic mutations in Taiwan Biobank participants: Data derived from the Axiom Genome-Wide CHB 1 and 2 Array plates with genotype imputation; Table S2: Association of *GCKR* rs143881585 genotypes with clinical phenotypes and laboratory parameters; Table S3: Association of *GCKR* rs143881585 genotypes with atherosclerotic risk factors; Table S4: Association of the *GCKR* rs8179206 genotypes with clinical phenotypes and laboratory parameters; Table S5: Association of the *GCKR* rs8179206 genotypes with atherosclerotic risk factors; Table S6: Association between *GCKR* rs1414321043 genotypes and metabolic and hematological phenotypes; Table S7: Association between *GCKR* rs1414321043 genotypes and atherosclerotic risk factors; Table S8. Association of the *GCKR* rs146175795 genotypes with metabolic and hematological phenotypes; Table S9. Association of the *GCKR* rs146175795 genotypes and atherosclerotic risk factors; Table s10: Association of the *GCKR* rs150673460 genotypes with metabolic and hematological phenotypes; Table S11: Association of the *GCKR* rs150673460 genotypes and atherosclerotic risk factors; Table S12: Association of the *GCKR* rs149847328 genotypes with metabolic and hematological phenotypes; Table S13: Association between *GCKR* rs149847328 genotypes and atherosclerotic risk factors; Table S14: Association of the *GCKR* rs146285804 genotypes with metabolic and hematological phenotypes; Table S15: Association of the *GCKR* rs146285804 genotypes and atherosclerotic risk factors; Table S16: Logistic regression analysis for metabolic syndrome, including genotypes, in 81,097 participants. Figure S1: Regional plot association studies genetic variants at positions 27.62 to 27.85 mega-base on chromosome 2p23.3 for systolic BP without (A) or with (B) conditional analysis; Figure S2: Regional plot association studies genetic variants at positions 27.62 to 27.85 mega-base on chromosome 2p23.3 for diastolic BP without (A) or with (B) conditional analysis; Figure S3: Regional plot association studies genetic variants at positions 27.62 to 27.85 mega-base on chromosome 2p23.3 for mean BP without (A) or with (B) conditional analysis; Figure S4: Regional plot association studies genetic variants at positions 27.62 to 27.85 mega-base on chromosome 2p23.3 for total cholesterol level without (A) or with (B) conditional analysis; Figure S5: Regional plot association studies genetic variants at positions 27.62 to 27.85 mega-base on chromosome 2p23.3 for serum low-density lipoprotein cholesterol level without (A) or with (B) conditional analysis; Figure S6: Regional plot association studies genetic variants at positions 27.62 to 27.85 mega-base on chromosome 2p23.3 for fasting plasma glucose level without (A) or with (B) conditional analysis; Figure S7: Regional plot association studies genetic variants at positions 27.62 to 27.85 mega-base on chromosome 2p23.3 for serum uric acid level without (A) or with (B) conditional analysis; Figure S8: Regional plot association studies genetic variants at positions 27.62 to 27.85 mega-base on chromosome 2p23.3 for serum creatinine levels without (A) or with (B) conditional analysis; Figure S9: Regional plot association studies genetic variants at positions 27.62 to 27.85 mega-base on chromosome 2p23.3 for estimated glomerular filtration rate without (A) or with (B) conditional analysis; Figure S10: Regional plot association studies genetic variants at positions 27.62 to 27.85 mega-base on chromo-some 2p23.3 for BUN without (A) or with (B) conditional analysis; Figure S11: Regional plot association studies genetic variants at positions 27.62 to 27.85 mega-base on chromosome 2p23.3 for albuminuria without (A) or with (B) conditional analysis; Figure S12: Regional plot association studies genetic variants at positions 27.62 to 27.85 mega-base on chromosome 2p23.3 for serum AST levels without (A) or with (B) conditional analysis; Figure S13: Regional plot association studies genetic variants at positions 27.62 to 27.85 mega-base on chromosome 2p23.3 for ALT without (A) or with (B) conditional analysis; Figure S14: Regional plot association studies genetic variants at positions 27.62 to 27.85 mega-base on chromosome 2p23.3 for serum γGT level without (A) or with (B) conditional analysis; Figure S15: Regional plot association studies genetic variants at positions 27.62 to 27.85 mega-base on chromosome 2p23.3 for leukocyte count without (A) or with (B) conditional analysis; Figure S16: Regional plot association studies genetic variants at positions 27.62 to 27.85 mega-base on chromosome 2p23.3 for platelet count without (A) or with (B) conditional analysis; Figure S17: Regional plot association studies genetic variants at positions 27.62 to 27.85 mega-base on chromosome 2p23.3 for the risk of hypertension without (A) or with (B) conditional analysis; Figure S18: Regional plot association studies genetic variants at positions 27.62 to 27.85 mega-base on chromosome 2p23.3 for the risk of gout without (A) or with (B) conditional analysis; Figure S19: Regional plot association studies genetic variants at positions 27.62 to 27.85 mega-base on chromosome 2p23.3 for the risk of microalbuminuria with-out (A) or with (B) conditional analysis; Figure S20: Regional plot association studies genetic variants at positions 27.62 to 27.85 mega-base on chromosome 2p23.3 for the risk of metabolic syndrome without (A) or with (B) conditional analysis; Figure S21: Linkage disequilibrium map of *GCKR* gene region rare mutations.
