# Supplementary material for: Pleiotropic Effects of Common and Rare GCKR Exonic Mutations on Cardiometabolic Traits
Source: Genes (Basel). 2022 Mar 10;13(3):491. doi: 10.3390/genes13030491 (PMC8951277; doi:10.3390/genes13030491)
Supplement: Supplementary file 1 [file genes-13-00491-s001.zip › genes-1594124-supplementary.pdf]

Supplementary Method 1.

Definitions of hypertension, diabetes mellitus, obesity, current smoking, microalbuminuria and metabolic syndrome

Hypertension was defined as systolic blood pressure (BP) of ≥140 mmHg, diastolic BP of ≥90 mmHg or a self-reported history of hypertension. Diabetes mellitus (DM) was defined as a fasting plasma glucose level of ≥126 mg/dL, a glycohemoglobin value of ≥6.5%, or a self-reported history of DM. Obesity was defined as a BMI of ≥25 kg/m^2. Current smoking was defined as regular cigarette smoking at the time of survey. Because of the absence of urine creatinine level, only spot urine albumin level was used for the evaluation of urine albumin. Microalbuminuria was defined as urine albumin of ≥30 mg/L.

Because medication histories were unavailable, metabolic syndrome characteristics were based on the recent update of the third report of the National Cholesterol Education Program's Adult Treatment Panel III criteria [1] with modifications. Participants with three or more of the following attributes are typically defined as having metabolic syndrome: (1) BP of ≥130/85 mmHg or a history of hypertension; (2) triglyceride level of ≥150 mg/dL; (3) high-density lipoprotein cholesterol level of <40 mg/dL for men or <50 mg/dL for women; (4) fasting plasma glucose of ≥100 mg/dL or a history of DM; and (5) waist circumference of >90 cm for men or >80 cm for women.

Reference:

1. Grundy SM, Cleeman JI, Daniels SR, Donato KA, Eckel RH, Franklin BA, et al. Diagnosis and management of the metabolic syndrome: an American Heart Association/National Heart, Lung, and Blood Institute Scientific Statement. Circulation. 2005; 11: 2735–52.

Supplementary Table S1. *GCKR* exonic mutations in Taiwan Biobank participants: Data derived from the Axiom Genome-Wide CHB 1 and 2 Array plates with genotype imputation

Chr	SNP	bp (GRCh38)	Ref/Alt	HWE	MAF	Exon number	Exonic Function	mRNA sequence	AA Change.
2	rs8179206	27497575	A / G	0.8864	0.0272	3	Non-synonymous mutation	c.A230G	p. Glu77Gly
2	rs146175795	27498276	G / A	0.3211	0.0065	4	Non-synonymous mutation	c.G307A	p. Val103Met
2	rs143881585	27498323	G / A	0.8717	0.0119	4	Synonymous, mutation	c.G354A	p. Ser118Ser
2	rs150673460	27498764	C / T	0.5407	0.0020	4	Non-synonymous mutation	c.C395T	p. Pro132Leu
2	rs149847328	27503548	C / T	0.9470	0.0002	9	Non-sense mutation	c.C679T	p. Arg227X
2	rs1414321043	27506551	G / T	0.8380	0.0007	11	Non-synonymous mutation	c. G940T	p. Ala314Ser
2	rs1260326	27508073	T / C	0.4474	0.4917	15	Non-synonymous mutation	c.T1337C	p. Leu446Pro
2	rs146285804	27518916	G / T	0.2744	0.0019	17	Non-synonymous mutation	c.G1551T	p. Trp517Cys

Chr: chromosome, SNP: single nucleotide polymorphism, bp: base pairs, Ref/Alt: reference/alternate allele, HWE: HWE: Hardy-Weinberg equilibrium, MAF: minor allele frequency, mRNA: messenger RNA, AA: amino acid.

Supplementary Table S2. Association of *GCKR* rs143881585 genotypes with clinical phenotypes and laboratory parameters

Clinical and laboratory parameters		beta	SE	*P* value*	*P* value**
Anthropology	Age (years)	0.1003	0.2442	0.6812	0.5546
	Waist circumference (cm)	-0.0111	0.1165	0.9241	0.8752
	Waist-hip ratio	0.0024	0.0012	0.0458	0.0347
	Body mass index (kg/m^2)	-0.0419	0.0830	0.6139	0.4739
Blood Pressure	Systolic BP* (mmHg)	0.4918	0.3495	0.1594	0.0430
	Diastolic BP* (mmHg)	0.3524	0.2279	0.1220	0.0377
	Mean BP* (mmHg)	0.3989	0.2504	0.1112	0.0276
Lipid profiles	Total cholesterol**** (mg/dL) (mmol/L)	0.0030	0.0018	0.0939	0.0015
	HDL-cholesterol**** (mg/dL) (mmol/L)	-0.0037	0.0022	0.0916	0.0774
	LDL-cholesterol**** (mg/dL) (mmol/L)	0.0024	0.0027	0.3795	0.0750
	Triglyceride**** (mg/dL) (mmol/L)	0.0327	0.0051	1.17×10^{-10}	3.69×10^{-22}
Glucose metabolism	Fasting plasma glucose** (mg/dL) (mmol/L)	0.0328	0.3451	0.9244	0.3939
	HbA1C** (%) (mmole/mol)	-0.0131	0.0138	0.3418	0.2653
Uric acid	Uric acid*** (mg/dL) (mmol/L)	0.0249	0.0256	0.3308	0.0140
Renal function	Creatinine (mg/dL) (mmol/L)	-0.0024	0.0049	0.6270	0.1999
	eGFR (mL/min/1.73 m^2)	0.6747	0.4945	0.1725	0.0102
	Urine albumin (mg/L)	0.0168	0.0104	0.1068	0.0180
Liver function	AST (U/L) (µkat/L)	0.5384	0.2770	0.0519	0.0073
	ALT (U/L) (µkat/L)	1.0851	0.4280	0.0112	0.0024
	γGT (U/L) (µkat/L)	1.5749	0.7106	0.0267	0.0012
	Serum albumin (g/dL) (µmol/ L)	0.0229	0.0051	7.00×10^{-6}	1.24×10^{-10}
	Total bilirubin (mg/dL) (µmol/ L)	0.0115	0.0061	0.0600	0.0377
Hematological parameters	Leukocyte count (10^3/µL)	0.0904	0.0352	0.0102	0.0007
	Hematocrit (%)	0.1327	0.0798	0.0962	0.1544
	Platelet count (10^3/µL)	-0.1899	1.3034	0.8842	0.4894
	Red blood cell count (10^6/µL)	-0.0197	0.0101	0.0514	0.0257
	Hemoglobin (g/dL)	0.0406	0.0282	0.1494	0.2179

Adjustments and participants recruited for analysis as in Fig 1.
*Adjusted for age, sex, BMI, and current smoking.
**Further adjusted for rs1260326 genotypes.
Abbreviations: GCKR, glucokinase regulator; SE, standard error; BP, blood pressure; HDL, high-density lipoprotein; LDL, low-density lipoprotein; HbA1C, hemoglobin A1C; eGFR, estimated glomerular filtration rate; BUN, blood urea nitrogen; AST, aspartate aminotransferase; ALT, alanine aminotransferase; γ GT, γ -Glutamyl transferase; BMI, body mass index.

Supplementary Table S3. Association of *GCKR* rs143881585 genotypes with atherosclerotic risk factors

	GG	GA	AA	beta	SE	*P1* value*	*P2* value**
Diabetes mellitus	9.5%	9.5%	0.0%	-0.0286	0.0828	0.7298	0.5914
Hypertension	22.4%	21.9%	23.1%	-0.0487	0.0613	0.4269	0.8230
Current smoking	9.1%	8.8%	15.4%	-0.0841	0.0842	0.3178	0.3605
Gout	3.9%	4.3%	7.7%	0.0525	0.1171	0.6538	0.3364
Microalbuminuria	11.3%	12.1%	30.8%	0.1040	0.0707	0.1414	0.0268
Metabolic syndrome	24.8%	26.4%	38.5%	0.1228	0.0599	0.0402	0.0040

*Adjusted for age, sex, BMI, and current smoking.
Current smoking: adjusted for age, BMI, and sex.
**Further adjusted for rs1260326 genotypes.

Supplementary Table S4. Association of the *GCKR* rs8179206 genotypes with clinical phenotypes and laboratory parameters

		beta	SE	*P1* value*	*P2* value**	*P3* value***
Anthropology	Age (years)	0.0398	0.1637	0.8080	0.7149	0.6885
	Waist circumference (cm)	0.0586	0.0781	0.4528	0.3517	0.3757
	Waist-hip ratio	-0.0001	0.0008	0.8678	0.9300	0.9092
	Body mass index (kg/m^2)	-0.0869	0.0556	0.1183	0.0839	0.0816
Blood Pressure	Systolic BP* (mmHg)	-0.0659	0.2356	0.7797	0.8790	0.8602
	Diastolic BP* (mmHg)	-0.0051	0.1536	0.9734	0.7312	0.7134
	Mean BP* (mmHg)	-0.0254	0.1688	0.8805	0.7800	0.7605
Lipid profiles	Total cholesterol**** (mg/dL) (mmol/L)	0.0022	0.0012	0.0686	0.0031	0.0037
	HDL-cholesterol**** (mg/dL) (mmol/L)	0.0019	0.0014	0.1944	0.2141	0.2532
	LDL-cholesterol**** (mg/dL) (mmol/L)	0.0013	0.0018	0.4785	0.1644	0.1952
	Triglyceride**** (mg/dL) (mmol/L)	0.0096	0.0034	0.0046	1.50×10^{-7}	3.89×10^{-8}
Glucose metabolism	Fasting plasma glucose** (mg/dL) (mmol/L)	-0.5149	0.2315	0.0261	0.0033	0.0026
	HbA1C** (%) (mmole/mol)	0.0000	0.0092	0.9964	0.9074	0.8527
Uric acid	Uric acid*** (mg/dL) (mmol/L)	0.0075	0.0171	0.6608	0.1216	0.1266
Renal function	Creatinine (mg/dL) (mmol/L)	-0.0020	0.0033	0.5498	0.2348	0.2167
	eGFR (mL/min/1.73 m^2)	0.3980	0.3315	0.2299	0.0362	0.0280
	Urine albumin (mg/L)	-0.0007	0.0070	0.9180	0.6574	0.5972
Liver function	AST (U/L) (µkat/L)	0.0528	0.1857	0.7764	0.4011	0.3996
	ALT (U/L) (µkat/L)	0.1913	0.2863	0.5041	0.2949	0.2992
	γGT (U/L) (µkat/L)	0.1644	0.4794	0.7316	0.2713	0.2314
	Serum albumin (g/dL) (µmol/ L)	0.0132	0.0034	1.19×10^{-4}	1.18×10^{-7}	3.11×10^{-8}
	Total bilirubin (mg/dL) (µmol/ L)	0.0047	0.0041	0.2543	0.2026	0.1754
Hematological parameters	Leukocyte count (10^3/µL)	-0.0218	0.0236	0.3568	0.7316	0.8184
	Hematocrit (%)	-0.0547	0.0535	0.3063	0.2270	0.2381
	Platelet count (10^3/µL)	1.1155	0.8750	0.2024	0.0576	0.0555
	Red blood cell count (10^6/µL)	0.0015	0.0068	0.8193	0.9814	0.9975
	Hemoglobin (g/dL)	0.0069	0.0189	0.7146	0.8422	0.8773

Adjustments and participants recruited for the analysis as in Fig 1.
*Adjusted for age, sex, BMI, and current smoking.
**Further adjusted for rs1260326 genotypes.
***Further adjusted for rs1260326 and rs143881585 genotypes.
Abbreviations as in supplementary Table 2

Supplementary Table S5. Association of the *GCKR* rs8179206 genotypes with atherosclerotic risk factors

	AA	GA	GG	beta	SE	P value*	*P2* value**	*P3* value***
Diabetes mellitus (%)	9.5	9.6	8.1	0.0224	0.0549	0.6835	0.7807	0.7825
Hypertension (%)	22.4	22.3	16.1	0.0037	0.0410	0.9278	0.6077	0.5415
Current smoking (%)	9.0	9.8	8.1	0.1044	0.0549	0.0572	0.0470	0.0398
Gout (%)	3.9	4.1	4.8	0.0953	0.0807	0.2374	0.1130	0.1309
Microalbuminuria (%)	11.3	11.5	11.3	0.0319	0.0487	0.5126	0.2361	0.2158
Metabolic syndrome (%)	24.9	24.8	24.9	0.0327	0.0409	0.4243	0.1601	0.1269

*Adjusted for age, sex, BMI, and current smoking.
Current smoking: adjusted for age, BMI, and sex.
**Further adjusted for rs1260326 genotypes.
***Further adjusted for rs1260326 and rs143881585 genotypes.

Supplementary Table S6. Association between *GCKR* rs1414321043 genotypes and metabolic and hematological phenotypes

Clinical and laboratory parameters		beta	SE	*P* value*
Anthropology	Age (years)	0.6573	1.0131	0.5165
	Waist circumference (cm)	-0.8136	0.4835	0.0924
	Waist-hip ratio	-0.0062	0.0051	0.2226
	Body mass index (kg/m^2)	0.3311	0.3443	0.3362
Blood Pressure	Systolic BP* (mmHg)	-3.4839	1.5377	0.0235
	Diastolic BP* (mmHg)	-0.9871	1.0025	0.3248
	Mean BP* (mmHg)	-1.8194	1.1016	0.0986
Lipid profiles	Total cholesterol**** (mg/dL) (mmol/L)	0.0084	0.0075	0.2611
	HDL-cholesterol**** (mg/dL) (mmol/L)	0.0025	0.0090	0.7856
	LDL-cholesterol**** (mg/dL) (mmol/L)	0.0079	0.0113	0.4813
	Triglyceride**** (mg/dL) (mmol/L)	0.0213	0.0211	0.3107
Glucose metabolism	Fasting plasma glucose** (mg/dL) (mmol/L)	-0.4794	1.4289	0.7372
	HbA1C** (%) (mmole/mol)	0.0138	0.0570	0.8086
Uric acid	Uric acid*** (mg/dL) (mmol/L)	0.1636	0.1046	0.1179
Renal function	Creatinine (mg/dL) (mmol/L)	-0.0062	0.0203	0.7612
	eGFR (mL/min/1.73 m^2)	0.6108	2.0518	0.7659
	Urine albumin (mg/L)	-0.0029	0.0433	0.9465
Liver function	AST (U/L) (µkat/L)	1.2083	1.1503	0.2935
	ALT (U/L) (µkat/L)	2.1065	1.7755	0.2355
	γGT (U/L) (µkat/L)	1.2968	2.9613	0.6615
	Serum albumin (g/dL) (µmol/ L)	0.0246	0.0212	0.2450
	Total bilirubin (mg/dL) (µmol/ L)	-0.0290	0.0254	0.2543
Hematological parameters	Leukocyte count (10^3/µL)	-0.0095	0.1461	0.9480
	Hematocrit (%)	-0.1980	0.3310	0.5498
	Platelet count (10^3/µL)	1.7015	5.4072	0.7530
	Red blood cell count (10^6/µL)	-0.0262	0.0419	0.5317
	Hemoglobin (g/dL)	0.0292	0.1168	0.8023

Adjustments and participants recruited for the analysis as in Fig 1.
*Adjusted for age, sex, BMI, and current smoking.
Abbreviations as in supplementary Table 2

Supplementary Table S7. Association between *GCKR* rs1414321043 genotypes and atherosclerotic risk factors

	GG	TG	beta	SE	*P* value*
Diabetes mellitus (%)	9.5%	12.6%	0.2080	0.3039	0.4937
Hypertension (%)	22.4%	29.7%	0.2904	0.2369	0.2204
Current smoking (%)	9.1%	5.4%	-0.6929	0.4324	0.1091
Gout (%)	3.9%	1.8%	-1.0512	0.7245	0.1468
Microalbuminuria (%)	11.3%	13.5%	0.1609	0.2816	0.5677
Metabolic syndrome (%)	22.2%	26.1%	0.1019	0.2477	0.6808

*Adjusted for age, sex, BMI, and current smoking.
Current smoking: adjusted for age, BMI, and sex.

Supplementary Table S8. Association of the *GCKR* rs146175795 genotypes with metabolic and hematological phenotypes

Clinical and laboratory parameters		beta	SE	*P* value*
Anthropology	Age (years)	-0.4926	0.3296	0.1351
	Waist circumference (cm)	-0.1277	0.1573	0.4167
	Waist-hip ratio	0.0017	0.0016	0.2988
	Body mass index (kg/m^2)	-0.1249	0.1120	0.2648
Blood Pressure	Systolic BP* (mmHg)	0.9074	0.4770	0.0571
	Diastolic BP* (mmHg)	0.4283	0.3110	0.1685
	Mean BP* (mmHg)	0.5880	0.3418	0.0854
Lipid profiles	Total cholesterol**** (mg/dL) (mmol/L)	0.0034	0.0025	0.1696
	HDL-cholesterol**** (mg/dL) (mmol/L)	-0.0065	0.0030	0.0275
	LDL-cholesterol**** (mg/dL) (mmol/L)	0.0044	0.0037	0.2370
	Triglyceride**** (mg/dL) (mmol/L)	0.0300	0.0069	1.50×10^{-5}
Glucose metabolism	Fasting plasma glucose** (mg/dL) (mmol/L)	-0.0397	0.4679	0.9324
	HbA1C** (%) (mmole/mol)	0.0057	0.0187	0.7613
Uric acid	Uric acid*** (mg/dL) (mmol/L)	0.0500	0.0345	0.1474
Renal function	Creatinine (mg/dL) (mmol/L)	-0.0092	0.0066	0.1646
	eGFR (mL/min/1.73 m^2)	1.3894	0.6675	0.0374
	Urine albumin (mg/L)	0.0225	0.0141	0.1094
Liver function	AST (U/L) (µkat/L)	0.2895	0.3744	0.4395
	ALT (U/L) (µkat/L)	-0.0169	0.5776	0.9766
	γGT (U/L) (µkat/L)	0.1060	0.9641	0.9124
	Serum albumin (g/dL) (µmol/ L)	0.0285	0.0069	3.50×10^{-5}
	Total bilirubin (mg/dL) (µmol/ L)	0.0005	0.0083	0.9478
Hematological parameters	Leukocyte count ($10^3/\mu L$)	-0.0258	0.0475	0.5876
	Hematocrit (%)	-0.3161	0.1077	0.0033
	Platelet count ($10^3/\mu L$)	2.4589	1.7612	0.1627
	Red blood cell count ($10^6/\mu L$)	-0.0116	0.0136	0.3940
	Hemoglobin (g/dL)	-0.0824	0.0380	0.0302

Adjustments and participants recruited for the analysis as in Fig 1.
*Adjusted for age, sex, BMI, and current smoking.
Abbreviations as in supplementary Table 2

Supplementary Table S9. Association of the *GCKR* rs146175795 genotypes and atherosclerotic risk factors

	GG	GA	AA	beta	SE	*P* value*
Diabetes mellitus (%)	9.5%	10.8%	50.0%	0.2378	0.1044	0.0227
Hypertension (%)	22.4%	23.4%	100%	0.1495	0.0811	0.0653
Current smoking (%)	9.1%	9.1%	0.0%	-0.0325	0.1130	0.7739
Gout (%)	3.9%	4.4%	0.0%	0.1322	0.1573	0.4006
Microalbuminuria (%)	11.3%	12.4%	0.0%	0.1350	0.0951	0.1555
Metabolic syndrome (%)	22.2%	24.7%	100%	0.2872	0.0822	0.0005

*Adjusted for age, sex, BMI, and current smoking.
Current smoking: adjusted for age, BMI, and sex.

Supplementary Table S10. Association of the *GCKR* rs150673460 genotypes with metabolic and hematological phenotypes

Clinical and laboratory parameters		beta	SE	*P* value*
Anthropology	Age (years)	0.3270	0.5947	0.5825
	Waist circumference (cm)	0.3579	0.2837	0.2072
	Waist-hip ratio	0.0049	0.0030	0.1000
	Body mass index (kg/m^2)	-0.0994	0.2021	0.6227
Blood Pressure	Systolic BP* (mmHg)	-0.5148	0.8494	0.5445
	Diastolic BP* (mmHg)	-0.6235	0.5538	0.2602
	Mean BP* (mmHg)	-0.5873	0.6086	0.3345
Lipid profiles	Total cholesterol**** (mg/dL) (mmol/L)	0.0085	0.0045	0.0575
	HDL-cholesterol**** (mg/dL) (mmol/L)	-0.0037	0.0054	0.4928
	LDL-cholesterol**** (mg/dL) (mmol/L)	0.0062	0.0067	0.3576
	Triglyceride**** (mg/dL) (mmol/L)	0.0526	0.0125	2.70×10^{-5}
Glucose metabolism	Fasting plasma glucose** (mg/dL) (mmol/L)	0.5406	0.8439	0.5217
	HbA1C** (%) (mmole/mol)	0.0396	0.0336	0.2386
Uric acid	Uric acid*** (mg/dL) (mmol/L)	0.0075	0.0631	0.9050
Renal function	Creatinine (mg/dL) (mmol/L)	0.0050	0.0119	0.6732
	eGFR (mL/min/1.73 m^2)	1.2388	1.2044	0.3037
	Urine albumin (mg/L)	0.0442	0.0254	0.0816
Liver function	AST (U/L) (μkat/L)	0.5700	0.6754	0.3987
	ALT (U/L) (μkat/L)	1.1304	1.0415	0.2778
	γGT (U/L) (μkat/L)	2.5694	1.7331	0.1382
	Serum albumin (g/dL) (μmol/ L)	0.0253	0.0124	0.0419
	Total bilirubin (mg/dL) (μmol/ L)	0.0310	0.0149	0.0382
Hematological parameters	Leukocyte count (10^3/μL)	0.1691	0.0857	0.0486
	Hematocrit (%)	0.1198	0.1943	0.5376
	Platelet count (10^3/μL)	1.5441	3.1781	0.6271
	Red blood cell count (10^6/μL)	0.0225	0.0246	0.3601
	Hemoglobin (g/dL)	0.0437	0.0686	0.5241

Adjustments and participants recruited for the analysis as in Fig 1.
*Adjusted for age, sex, BMI, and current smoking.
Abbreviations as in supplementary Table 2

Supplementary Table S11. Association of the *GCKR* rs150673460 genotypes and atherosclerotic risk factors

	CC	CT	beta	SE	*P* value*
Diabetes mellitus (%)	90.5%	89.8%	0.0945	0.1956	0.6289
Hypertension (%)	22.4%	21.1%	-0.0851	0.1512	0.5735
Current smoking (%)	9.1%	9.3%	0.1983	0.2331	0.3949
Gout (%)	3.9%	7.1%	0.7106	0.2321	0.0022
Microalbuminuria (%)	11.3%	12.1%	0.0804	0.1738	0.6437
Metabolic syndrome (%)	22.2%	22.6%	0.0804	0.1738	0.6437

*Adjusted for age, sex, BMI, and current smoking.
Current smoking: adjusted for age, BMI, and sex.

Supplementary Table S12. Association of the *GCKR* rs149847328 genotypes with metabolic and hematological phenotypes

Clinical and laboratory parameters		beta	SE	*P* value*
Anthropology	Age (years)	2.2046	1.7538	0.2087
	Waist circumference (cm)	1.0107	0.8371	0.2273
	Waist-hip ratio	0.0076	0.0087	0.3836
	Body mass index (kg/m^2)	1.0528	0.5960	0.0773
Blood Pressure	Systolic BP* (mmHg)	-2.1274	2.7589	0.4407
	Diastolic BP* (mmHg)	-0.3234	1.7985	0.8573
	Mean BP* (mmHg)	-0.9248	1.9765	0.6399
Lipid profiles	Total cholesterol**** (mg/dL) (mmol/L)	0.0039	0.0130	0.7636
	HDL-cholesterol**** (mg/dL) (mmol/L)	-0.0006	0.0156	0.9687
	LDL-cholesterol**** (mg/dL) (mmol/L)	0.0148	0.0195	0.4468
	Triglyceride**** (mg/dL) (mmol/L)	0.0160	0.0365	0.6604
Glucose metabolism	Fasting plasma glucose** (mg/dL) (mmol/L)	-2.8907	2.5214	0.2516
	HbA1C** (%) (mmole/mol)	-0.0604	0.1005	0.5481
Uric acid	Uric acid*** (mg/dL) (mmol/L)	0.0789	0.1961	0.6873
Renal function	Creatinine (mg/dL) (mmol/L)	0.0146	0.0352	0.6789
	eGFR (mL/min/1.73 m^2)	-2.5759	3.5523	0.4684
	Urine albumin (mg/L)	0.1967	0.0749	0.0087
Liver function	AST (U/L) (µkat/L)	-1.9899	1.9918	0.3178
	ALT (U/L) (µkat/L)	-3.4733	3.0736	0.2585
	γGT (U/L) (µkat/L)	0.5825	5.1284	0.9096
	Serum albumin (g/dL) (µmol/ L)	0.0329	0.0367	0.3705
	Total bilirubin (mg/dL) (µmol/ L)	-0.0462	0.0440	0.2942
Hematological parameters	Leukocyte count (10^3/µL)	0.0614	0.2529	0.8083
	Hematocrit (%)	-0.8675	0.5731	0.1301
	Platelet count (10^3/µL)	5.6052	9.3713	0.5498
	Red blood cell count (10^6/µL)	0.0365	0.0725	0.6145
	Hemoglobin (g/dL)	-0.1769	0.2022	0.3818

Adjustments and participants recruited for the analysis as in Fig 1.
*Adjusted for age, sex, BMI, and current smoking.
Abbreviations as in supplementary Table 2

Supplementary Table S13. Association between *GCKR* rs149847328 genotypes and atherosclerotic risk factors

Genotypes	CC	TC	beta	SE	*P* value*
Diabetes mellitus (%)	9.5%	16.2%	0.2804	0.4836	0.5620
Hypertension (%)	22.4%	27.0%	-0.1478	0.4281	0.7299
Current smoking (%)	9.1%	5.4%	-0.5701	0.7488	0.4465
Gout (%)	3.9%	16.2%	1.4906	0.5170	0.0039
Microalbuminuria (%)	11.3%	18.9%	0.4629	0.4269	0.2782
Metabolic syndrome (%)	22.2%	27.0%	-0.1711	0.4305	0.6910

*Adjusted for age, sex, BMI, and current smoking.
Current smoking: adjusted for age, BMI, and sex.

Supplementary Table S14. Association of the *GCKR* r rs146285804 genotypes with metabolic and hematological phenotypes

Clinical and laboratory parameters		beta	SE	*P* value*
Anthropology	Age (years)	-0.1566	0.6061	0.7962
	Waist circumference (cm)	-0.2911	0.2891	0.3139
	Waist-hip ratio	-0.0048	0.0030	0.1121
	Body mass index (kg/m^2)	-0.0017	0.2059	0.9936
Blood Pressure	Systolic BP* (mmHg)	1.1218	0.8660	0.1952
	Diastolic BP* (mmHg)	0.4826	0.5649	0.3929
	Mean BP* (mmHg)	0.6957	0.6206	0.2623
Lipid profiles	Total cholesterol**** (mg/dL) (mmol/L)	0.0076	0.0045	0.0911
	HDL-cholesterol**** (mg/dL) (mmol/L)	0.0019	0.0054	0.7247
	LDL-cholesterol**** (mg/dL) (mmol/L)	0.0099	0.0067	0.1399
	Triglyceride**** (mg/dL) (mmol/L)	0.0196	0.0125	0.1178
Glucose metabolism	Fasting plasma glucose** (mg/dL) (mmol/L)	-0.6971	0.8572	0.4161
	HbA1C** (%) (mmole/mol)	-0.0539	0.0342	0.1150
Uric acid	Uric acid*** (mg/dL) (mmol/L)	0.0926	0.0628	0.1404
Renal function	Creatinine (mg/dL) (mmol/L)	0.0075	0.0122	0.5393
	eGFR (mL/min/1.73 m^2)	-0.3243	1.2272	0.7916
	Urine albumin (mg/L)	-0.0213	0.0259	0.4113
Liver function	AST (U/L) (μkat/L)	0.3561	0.6886	0.6050
	ALT (U/L) (μkat/L)	-0.1227	1.0614	0.9080
	γGT (U/L) (μkat/L)	-0.6704	1.7729	0.7053
	Serum albumin (g/dL) (μmol/ L)	0.0029	0.0127	0.8214
	Total bilirubin (mg/dL) (μmol/ L)	0.0029	0.0152	0.8497
Hematological parameters	Leukocyte count (10^3/μL)	0.0030	0.0874	0.9724
	Hematocrit (%)	0.0097	0.1981	0.9611
	Platelet count (10^3/μL)	-5.6662	3.2367	0.0800
	Red blood cell count (10^6/μL)	-0.0118	0.0251	0.6381
	Hemoglobin (g/dL)	0.0218	0.0699	0.7545

Adjustments and participants recruited for the analysis as in Fig 1.
*Adjusted for age, sex, BMI, and current smoking.
Abbreviations as in supplementary Table 2

Supplementary Table S15. Association of the *GCKR* r rs146285804 genotypes and atherosclerotic risk factors

Genotypes	GG	TG	beta	SE	*P* value*
Diabetes mellitus (%)	9.5%	8.7%	-0.0579	0.2111	0.7839
Hypertension (%)	22.4%	23.2%	0.1052	0.1487	0.4793
Current smoking (%)	9.1%	7.7%	-0.1396	0.2233	0.5318
Gout (%)	3.9%	2.6%	-0.3260	0.3676	0.3752
Microalbuminuria (%)	11.3%	9.0%	-0.2515	0.2008	0.2103
Metabolic syndrome (%)	22.2%	20.9%	-0.0544	0.1606	0.7346

*Adjusted for age, sex, BMI, and current smoking.
Current smoking: adjusted for age, BMI, and sex.

Supplementary Table S16. Logistic regression analysis for metabolic syndrome, including genotypes, in 81,097 participants

	OR (95%, CI)	*P* value
Age (years)	1.0619 (1.0598 - 1.0640)	$< 10^{-307}$
Sex (male *vs*. female)	1.1016 (1.0572 - 1.1479)	4.13×10^{-06}
Body mass index (kg/m^2)	1.3936 (1.3851 - 1.4022)	$< 10^{-307}$
Current smoking (%)	1.6919 (1.5861 - 1.8048)	2.32×10^{-57}
rs1260326 (TT *vs*. TC *vs*. CC)	1.0880 (1.0587 - 1.1181)	1.43E $\times 10^{-09}$
WGRS-GCKR*	3.3317 (2.0323 - 5.4620)	1.83×10^{-06}

WGRS-GCKR: weighted genetic risk scores derived from the combination of *GCKR* rs143881585 and rs1461755795 variants
